# Pairwise structural comparison of tiagabine analogs gives new insights into their protein binding modes

**DOI:** 10.1186/1758-2946-5-S1-P32

**Published:** 2013-03-22

**Authors:** Barbara Zdrazil, Andreas Jurik, Harald H Sitte, Gerhard F Ecker

**Affiliations:** 1 University of Vienna, Department of Medicinal Chemistry, Althanstrasse 14, Vienna, A-1090, Austria; 2 Medical University of Vienna, Institute of Pharmacology, Vienna, A-1090, Austria

## 

Tiagabine (Gabitril^®^) is a selective inhibitor of the human gamma-aminobutyric acid (GABA) transporter 1 (hGAT-1), a transport protein belonging to the family of neurotransmitter-sodium-symporters (NSS). It is a marketed drug, used for treatment of epilepsy. However, the molecular basis of protein-ligand interaction remains obscure due to the lack of a 3D structure of the target protein.

In order to identify activity-determining structural features of a series of tiagabine analogs taken from literature [1-3], we chose an approach combining traditional methods of molecular modeling with exhaustive sampling of docking poses, and a pairwise comparison of structural features and their respective bioactivity values.

We determined a common binding mode of tiagabine analogs, which is in nice agreement with literature [4]. Further, we were able to trace back considerable differences in inhibitory activities to distinct molecular attributes of the analogs.

Our study revealed the molecular explanation for the importance of a polar linker region and thus paves the way for subsequent screening efforts in the search for novel GAT-1 inhibitors.

## References

[B1] Andersen KE The synthesis of novel GABA uptake inhibitors. 1. Elucidation of the structure-activity studies leading to the choice of (R)-1-[4,4-bis(3-methyl-2-thienyl)-3-butenyl]-3-piperidinecarboxylic acid (tiagabine) as an anticonvulsant drug candidate J Med Chem 1993 36 1716 1725 10.1021/jm00064a005 8510100

[B2] Knutsen LJ Synthesis of novel GABA uptake inhibitors. 3. Diaryloxime and diarylvinyl ether derivatives of nipecotic acid and guvacine as anticonvulsant agents J Med Chem 1999 42 3447 3462 10.1021/jm981027k 10479278

[B3] Andersen KE Synthesis of novel GABA uptake inhibitors. 4. Bioisosteric transformation and successive optimization of known GABA uptake inhibitors leading to a series of potent anticonvulsant drug candidates J Med Chem 1999 42 4281 4291 10.1021/jm980492e 10543872

[B4] Skovstrup Homology modelling of the GABA transporter and analysis of tiagabine binding Chem Med Chem 2010 5 986 1000 20491137 10.1002/cmdc.201000100

